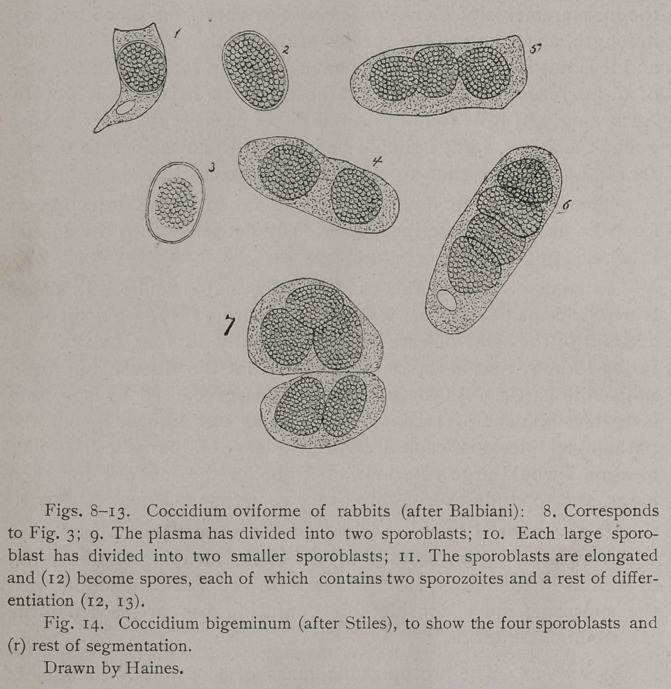# Notes on Parasites

**Published:** 1892-05

**Authors:** C. W. Stiles

**Affiliations:** B. A. I., U. S. Dept. of Agriculture, Washington, D. C.


### NOTES ON PARASITES.

C. W. Stiles, A. M., Ph. D.

### *10.—A Case of Intestinal Coccidiosis in Sheep.*

Leuckart mentions the sheep as one of the animals in which Coccidia are found inhabiting the epithelial cells of the intestines (Die Parasiten des Menschen I., p. 282), stating at the same time that he is personally acquainted with the intestinal Coccidia only of dogs and cats. In the literature at my disposal I have been unable to find the source from which Leuckart took his statement. Since the appearance of the work cited, several authors have quoted Leuckart as authority for the occurrence of this parasite in sheep, while others (Pfeiffer, in his second edition of Die Protozoen als Krankheitserreger, 1891,) omit the mention of sheep as one of the possible hosts for Coccidia.

This uncertainty in the minds of some authors leads me to describe a case of intestinal Coccidiosis in sheep, which *Dr. Curtice found about four years ago. The pathological lesions were at that time diagnosed by Dr. Theobald Smith as Coccidiosis, and the specimen was placed in the collection of parasites connected with the Bureau of Animal Industry. No history is connected with the specimen ; but Dr. Smith, in private conversation with me, described the original appearance of the intestines as follows : "On the mucosa of the small intestine there appeared irregular, slightly elevated, whitish patches, which on superficial observation gave the impression of a thin, spreading mycelium of some fungus. These patches were from three-fourths to one inch in diameter. When a portion of such a spot was examined fresh under the microscope, the intestinal villi were found very much enlarged. The epithelial cells lining these villi were very large, and every one contained one or more Coccidia cysts with distinct membrane and uniformly and coarsely granular contents. The change in the mucosa was thus entirely due to the invasion of the epithelium by Coccidia."

The specimen as I found it had been in alcohol four years, and had lost its original microscopic appearance; but a microscopic examination of the cells still showed the parasites very plainly.

In the patches described above, every epithelial cell contained one or more parasites, the greatest number of parasites found in

* Jour. Comp. Med. and Vet. Arch., April, 1892.

one cell being four (Fig. 6). Some of the cells were greatly distorted in shape, the nuclei being crowded to one side or to one end.

Figs. 1–7. Sporozoa (probably *Coccidium perforans*) from the intestinal epithelium of a sheep. 1. An epithelial cell containing a single parasite; 2. Free parasite; 3. The plasma has receded from the cyst wall, cf. Fig. 8; 5–7. Two to four parasites in each cell. Original drawn by Haines after sketches by the author.

The parasites were all nearly in the same stage of development, and consisted of a coarsely granular body with or without a double contoured membrane. One Coccidium was found in which the granular contents had receded from the cyst-wall (Fig. 3). This, however, might possibly have been caused by the action of the alcohol.

The parasites measured :

| | | | | |
|---|---|---|---|---|
| MM. 0.018 | long | by 0.015 | broad. | |
| 0.019 | " | 0.016 | " | |
| 0.021 | " | 0.015 | " | |

In using the term Coccidia above, I intend only to signify that the parasites described belong to the order Coccidia, since it is impossible to state positively that they belong to the genus *Coccidium*, a matter which can be settled only when other stages are found. Should it be found that the granular contents divide into sporoblasts (Fig. 10) the parasite will be a member of the genus *Cocidium.*

The intestinal Coccidium of rabbits and man belongs to the species *C. perforans*, Lkt., 1879,* and it is possible that the sheep parasite belongs to the same species, although it must be noted that the measurements given above do not agree with the measurements given for *C. perforans* (0.024 mm. long by 0.012 broad, after Reincke and Neumann; 0.026 to 0.035 long by 0.014 to 0.02, after Railliet and Lucet). Railliet and Lucet have, however, shown that

* It will be remembered that some authors (Leuckart, Railliet, etc.,) consider this form as specifically distinct from the liver Coccidium (*C. oviforme*), while other authors (L. Pfeiffer) look upon both forms as one species.

the measurements of *Coccidium bigeminum* vary from 0.008 to 0.015 in length, according to the species of mammal in which it is found, and it is not impossible that we have a similar variation in the size of *C. perforans* in the different host-species in which it occurs. Accordingly, if it be found that our ovine parasite belongs to the genus *Coccidium*, Lkt., I should be inclined to unite it with *C. perforans*, Lkt., rather than to make a new species of it.

There can be no doubt that such an enormous infection of Coccidia as is here described is seriously detrimental to the animal in which it occurs.

In connection with this case of intestinal coccidiosis in sheep it will be remembered that Pröger and Zürn (1877) published an account of the same disease resulting fatally in four calves. So far as I know, that is the only occasion that the disease has been noticed in cattle. As the subject of diseases caused by sporozoa is a development of recent years, and as our knowledge of the pathological lesions caused by this class of parasites is very meagre, I would urge veterinarians to be on the lookout for such cases, and to study thoroughly every case found.

In this paper I have made use of the term "sporoblast," and wish now to add a few lines in regard to the meaning of this term, as well as of the terms "*spore*," "*sporocyst*," etc., in connection with the sporozoa, since considerable confusion has arisen from the misuse of these terms.

*Sporoblast:* The protoplasmic mass of Coccidium, such as is represented in Figs. 1, 2, 8, afterward recedes from the surrounding membrane, and divides into two more or less circular bodies (Fig. 9); each of these bodies then divides into two smaller round bodies (Fig. 10). These four round bodies have been named "*sporoblasts*" by most authors. Pfeiffer used the same term in the first edition of his work, "Die Protozen als Krankheitserreger," 1890; but in his second edition (1891), following the nomenclature which Wolters uses in speaking of the *Gregarina*, he changes the name to "*sporogonie*."

*Spore:* When the "*sporoblasts*" become elongated and form a more or less distinct membrane of their own (Figs. 11, 12, 13), they are named "spores" by most authors. Pfeiffer again following Wolters, applies the name "*sporocyst*" to designate this stage, while Neumann broadens the application of the word "*sporoblast*" so that it includes the "*spore*" as well as the "*sporoblast*" of other authors.

The entire protoplasmic substance of the parasite is not used in the formation of the four "*sporoblasts*," but a small portion frequently remains (Fig. 14), to which the names "*reliquat de segmentation*" (Railliet et Lucet), "Theilungskorper" (German authors) have been applied, for which Pfeiffer uses "*sporophor*." But Wolters introduced the term "*sporophor*" in speaking of "*noyau de reliquat*," a small portion of protoplasma which is left unused in the "*macrospores*" and "*microspores*" (*pseudonaricellæ*) of the *Gregarina*, after the "*falciform bodies*" are formed. Wolters uses the terms "*sporocyst*" as equivalent to "*macrospore*" and "*microspore* of Gregarines, and the term "*spore*" as equivalent to "falciform body."

*Falciform bodies:* Every "*spore*" of the true *Coccidia* gives rise to two "*falciform bodies*" (Fig. 13), for which Thélohan sometimes uses the term "*sporozoites*," while Neumann and others call them "*spores*."

When the two "*falciform bodies*" are formed, a small portion of the plasma of the spore is left unused, and this has been called the "*nucleus*" or *noyau de reliquat*," or "*reliquat de differteniation*," or "Restkorper;" Pfeiffer uses the term "*sporophor*" to signify this body also. Strictly speaking, the "*sporophor*" of Wolters is homologous to this body.

It is unfortunate that these terms are so intermixed, and that new terms are introduced to designate forms which were well named years ago.

The following table (Part I) will show most of the names applied by authors to the different stages in the development of *Gregarines* and *Coccidia*. It will be immediately apparent that the same terms have been used by different authors—in fact, by the same author sometimes—to represent different stages of the same animal. *Part II shows the terms used in mycology to designate analogous stages of plants. Part III represents the new nomenclature of Wolters for the *Gregarina*, which Pfeiffer has adapted for the *Coccidia*. Part IV, which is essentially the nomenclature followed by most zoologists at present, gives the terms to which in my opinion we should adhere, at least until we are in a position to revise the nomenclature of the entire group of *Sporozoa*.

In regard to the "Wolters nomenclature," it will be apparent to all, I believe, that the term "*sporogonie*" has no advantage over the old term "*sporoblast*," while the term "*sporocyst*" not only has no advantage over the terms "*macrospore*" and "*microspore*," but is a particularly unhappy selection, since this name "*sporocyst*" is an every-day technical term, used by zoologists to denote a certain larval stage of flukes (Trematode worms), a larval stage which is neither analogous nor homologous to the "spores" (pseudonavicellæ) of the gregarines, for the "sporocyst" of *Trematodes* is a stage in the *ascending* series of development, *i. e.*, between the germ (ovum) and the adult, while the "spore" ("sporocyst" of Wolters) of the *Sporozoa* is in the *descending* series, *i. e.*, a stage between the adult and the germ which gives rise to the next generation.

Furthermore, there seems to be no reason for dropping the term "spore," since the stage thus named is actually analagous to the "spore" (conidium, zoosporangium) of the *Peronosporeæ*, so far as analogy can be drawn between these two groups, and is exactly analogous with the "spore" (zoosporangium) of the *Synchytriæ*. Moreover, the class *Sporozoa* was so named on account of the peculiar reproduction in this group of animals by means of "spores"

* I am indebted to Dr. Erwin T. Smith for checking Part II.

| I. Authors | | II. Analogous stages in plants (after DeBary, Zopf, Fisher, checked by Dr. E. F. Smith.) | | III. Wolters' Nomenclature. | | IV. Conclusion. | |
|---|---|---|---|---|---|---|---|
| | | | | Wolters. | L. Pfeiffer. | | |
| Gregarines. | Coccidia. | Peronosporeæ. | Synchytriæ. | Gregarines. | Coccidia. | Gregarines. | Coccidia. |
| Adult, (cephalin sporadin.) | Adult. | Myceilum. | Mycelium. | | | Adult (cephalin, sporadin). | Adult. |
| Cyst. | Cyst. | | Sorus. | Cyst. | Makrospore, Mikrospore. | Cyst. | Cyst. |
| | Filiment suspensur in Orthospora propria (Schneider). | Sporophor. | | | | | *Filiment suspenseur in Orthospora propria. |
| Sporoblast. | Sporoblast. | | | Sporogonie. | Sporogonie. | Sporoblast. | Sporoblast. |
| Spore, Pseudonavicelle, Navicelle, Macrospore, Microspore, Psorosperm. | Spore, Sporoblast (Neumann). | Conidium, (Gonidium) Spore. Zoosporangium. | Zoosporangium. | Sporocyst. | Sporocyst. | Spore, Pseudonavicelle, Macrospore, Microspore. | Spore. |
| Plasmamass, Pseudokyst, Plasmarest. | Reliquat de segmentation, noyau, de reliquat. | | | | Sporophor. | Pseudocyst, Plasmarest. | Rest of segmentation. |
| Falciform body, Falciform spore, | Falciform body, Falciform spore, Spore, Sporozoite. | Zoospore. | Zoospore. | Spore. | Sichelkeim, (Spore). | Sporozoite. Falciform body. | Sporozoite, Falciform body. |
| Nucleus or noyau de reliquat, Globule central, Restkorper. | Nucleus or noyau de reliquat, Rest of Differentiation, Restkorper. | | | Sporophor. | Sporophor. | Globule central, Restkorper. | Rest of differentiation, Restkorper. |
| Adult. | Adult. | Germ-tube, Mycelium. | Mycelium. | | | Adult. | Adult. |

* *i. e.*, a Sporoblastosphore.

(*i. e.*, pseudonavicelles and "spores" as the word is used in this paper), and not on account of the falciform bodies (sporozoites, "spores" of Wolters).

The term "*sporophor*" of Wolters must be looked upon as a miscarriage in the sense that it is used : *First*, because it has no analogy with the "*sporophor*" of fungi ; *secondly*, because a "*sporophor*" could, of course, bear only "spores," while the "*sporophor*" in the new sense is brought into relation with bodies which are not "spores" in the unqualified meaning of the term, but with the *sporozoites* (zoospores of fungi, *i. e.*, a qualified "spore"); *thirdly*, because the "*nucleus de reliquat*" does not bear, either in the sense of giving rise to or carrying the sporozoites, but is a portion of the plasma which, according to most authors, remains after the formation of the *sporozoites*, a view of which must now be modified, according to the investigations of Henneguy, who says: "Its presence in the spore, *before* the production of the falciform bodies, indicates that it does not result, as is ordinarily supposed, from the substance left unused in the formation of these spores; as its origin and evolution are not yet well understood, it would be better, I believe, to designate it as *globule central*."

Pfeiffer's application of Wolter's *sporophor* to the Coccidia is certainly illogical, since he uses it to represent two entirely different things, *i. e.*, the *rest of segmentation* and the *rest of differentiation*. If the term "*sporophor*" is retained at all, it should be applied to Schneider's "*filiment suspenseur*" in *Orthospora propria* (Schneider).

Pfeiffer's terms, "*makrospore*" and "*mikrospore*" should probably read "makrocyst" and "mikrocyst."

In the foregoing discussion I have intentionally omitted mention of the newly discovered mode of reproduction in *Coccidium*, in which the parasite does not divide into four spores, but gives rise directly to numerous swarmspores. The falciform bodies or swarmspores would of course be analogous to the sporozoites.

Pfeiffer compares this stage to *Eimeria*, and assumes that the "spore" stage (sporogonienstadium, Pfeiffer) of *Eimeria* is entirely lost. Judging from the figures of *Eimeria*, this is incorrect, for the plasma recedes from the wall and forms a second membrane around itself, *thus forming a spore*, while accordiug to Pfeiffer's figures there is but one membrane around *Coccidium oviforme* in this stage. More thorough investigation is necessary in regard to this point.

B. A. I., U. S. Dept. of Agriculture,
Washington, D. C., IV, 21, '92.

## Figures and Tables

**Figs. 1-7. f1:**
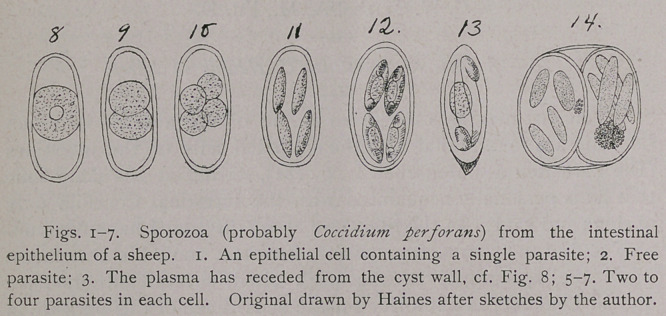


**Figs. 8-13. Fig. 14. f2:**